# Female education and maternal health care utilization: evidence from Uganda

**DOI:** 10.1186/s12978-022-01432-8

**Published:** 2022-06-20

**Authors:** David Amwonya, Nathan Kigosa, James Kizza

**Affiliations:** Department of Economics and Statistics, Kyambogo University, Kyambogo, P.O.BOX 1, Kampala, Uganda

**Keywords:** RDD, OLS, Fixed effects, 2SLS, MHC

## Abstract

**Background:**

Maternal health care is among the key indicators of population health and economic development. Therefore, the study attempted to explore female education and maternal healthcare utilization in Uganda. The study identified the causal effect of introduction of free education by exploiting the age as an instrument at the second stage model (BMC Health Serv Res. 2015. 10.1186/s12913-015-0943-8; Matern Child Health J. 2009;14:988–98). This instrument provided an exogenous source of variation in the years of schooling and allowed to implement a regression discontinuity design which accounted for heterogeneity in the cohort overtime.

**Methods:**

The study used the Ordinary Least Squares (OLS) to help predict years of schooling that were used in the second stage model in the Two Stage Least Squares (2SLS). The study further used the Regression Discontinuity Design (RDD) model with a running variable of birth years to observe its effect on education. To control for heterogeneity in regions in the second stage model, a fixed effects model was used.

**Results:**

Female education indeed had a positive impact on maternal health care utilization. It was further found out that age also influences maternal health care utilization.

**Conclusions:**

Therefore, as an effort to improve professional maternal health care utilisation, there is need to focus on education beyond primary level. Uganda Government should also ensure that there is an improvement in community infrastructure and security across all regions and locations.

## Plain Language summary

Maternal health care (MHC) utilization is one of the Millennium Development Goals (SDG) of pursuit. Globally, most low-income countries like Uganda contribute greatly to pregnancy-related mortalities that are largely preventable through adequate utilization of essential maternal health care services. Though Uganda over time has registered some increase in maternal utilization, this has been attributed to a number of factors. This study intended to demonstrate whether the introduction of free primary education in Uganda led to increase in the utilization of maternal health services. To address this, we used Ordinary Least Squares (OLS), Two Stage Least Squares (2SLS), Probit and Regression Discontinuity Design (RDD) models using Demographic Health Survey (DHS) data 2006 and 2011. The study found out that indeed the introduction of free primary education increased the utilization of MHC.

## Introduction and context

Maternal Health Care (MHC) is a core health outcome indicator as well as a persistent challenge to social-economic transformation of the developing world [1]. The inability to access MHC causes the Maternal Mortality Rate (MMR) to stay high especially in poor countries. The majority of these deaths are caused by health conditions that are preventable if detected early among pregnant mothers. The United Nations Sustainable Development Goals aim to reduce the maternal mortality to 70 deaths per 100,000 live births by 2030 [2]. As such, many parties including economists, health experts and government policy makers are interested in understanding the determinants of MHC utilization.

The wider socio-economic determinants of health are also important for the survival and wellbeing of a pregnant woman. According to the UN [3–6], these factors include economic conditions, education, culture, among others. Subsequently, this article studies a specific social determinant of MMR whose causality link has not been properly studied for the case of Uganda’s education. This study focuses on the education sector reform introduced in Uganda in 1997 to waive all school attendant costs for four primary school pupils per household of which two had to be girls. The reform was introduced due to the high cost of education and high numbers of school dropouts. This reform was not new as many developed countries provided free education to their children. In Sub Saharan Africa (SSA), Uganda was among the first few countries to adopt the reform among countries such as Malawi, and Ethiopia.

Some studies have found a positive relationship between maternal education and utilization of MHC [7–9]. However, for the case of Uganda, most existing studies are unable to explain whether the link between education and maternal health is attributable to other correlated factors such as economic growth and household income among others. Therefore, the study explains whether increasing women’s education indeed improves their health outcomes by exploiting an exogenous source of variation in education, in this case policy change for free primary education. The underlying hypothesis is that educated women utilize MHC services often more than the uneducated ones.

There are a wide range of social factors that determine the utilisation of MHC services and therefore impacting on the Maternal Mortality Ratio (MMR). Access to MHC is an ingredient to a wide range of health outcomes including mother morbidity and mortality, childcare and infant mortality, fertility, life expectancy etc. Like the rest of the developing world, Uganda still ranks high in the MMR [10]. According to the World Bank, this ratio has been falling from 580 deaths out of 100,000 live births in the year 2000 to 343 out of 100,000 births in 2018. Notwithstanding the above progress, the country still ranks among the top 40 countries in the world with high maternal mortality in comparison to the global target of 70 deaths per 100,000 live births [2]. Several studies have hypothesised potential mechanisms through which lack of education particularly among women affects their ability to utilize MHC services [10–14].

The aim of this article is to employ a cross-sectional study in one of the SSA Countries wherein, MMR is still high. Therefore, the study focusses solely on Uganda for this investigation. Like the rest of the developing world, Uganda still ranks high in MMR. The largest causes of MMR are issues related to complications during pregnancy such as blood pressure mainly attributed to short birth spacing and unwanted pregnancies. Also, some bad cultural practices and traditions that deter pregnant mothers from seeking MHC has been attributed to MMR.

The contribution that this article offers to the existing literature on education and MHC utilization is the unique context in which it is set. The ability to isolate education from other demographic and social factors which may be also highly correlated to education making it the first of its kind in the case of Uganda. Furthermore, although the study initially runs a 2 stage OLS regression, the key methodology employed here is the RDD. All existing studies have used other methodologies such as logistic and probit regressions to analyse the relationship between female education and MHC utilization but have never isolated other factors that have been found to be strongly related with education such as the rate of economic growth, household incomes, electricity access etc.

The question regarding the positive relationship between female education and MHC utilization is of great importance as it deepens our knowledge on the determinants of fertility, child health outcomes and life expectancy. These social demographic challenges are still prominent in SSA whose countries are below the world average [15]. In this sense, reduced MMR levels are likely to have positive impacts on society and children survival.

## Determinants of maternal healthcare utilization

Historically, like any other developing country in the SSA region, Uganda has had a very high maternal mortality rate although this rate has been falling gradually in the last 20 years [14, 16, 17]. The most common causes of maternal mortality in developing countries are pregnancy-related complications [18]. For the case of Uganda, according to the joint trend analysis report from 2000 to 2017 by WHO, UNICEF and World Bank, the high maternal related deaths have been aggravated by short birth spacing and high fertility levels (> = 5 births as at 2016). Fortunately, if detected in advance, these risk factors can be managed by healthcare professionals before they become fatal.

Several studies attribute utilization of maternal healthcare services to demographic-social factors at an individual, household and community level but emphasize the issue of selected areas such as districts, urban or rural or even selected countries [6, 19–22]. Most of such studies have had a localized selection bias generated through limiting the analysis on districts, regions, urban or rural areas and selected countries particularly in Sub-Saharan Africa instead of looking at broader cross cutting determinants of maternal healthcare utilization beyond locality [4, 23]. In order to do away with such a bias, they use the 2006 national Demographic Health Survey (DHS) data and examine the effect of age, place of residence, marital status, education, religion, birth order and wealth index on the utilization of maternal care services in Uganda by measuring maternal healthcare utilization in three categories i.e. the visits to antenatal clinic, tetanus toxoid injection and place of delivery.

Using the binary logistic regression model, it is confirmed that a place of residence indeed has a strong positive effect on maternal healthcare utilisation [4]. When they control for local fixed effects, they find that education, birth order, and wealth index are strongly associated with all the three categories or measures of maternal health care services, while age, marital status and religion were either moderately or insignificantly associated with maternal health care services. For instance, they find that 62.6% urban women had visited antenatal care service more than 4 times compared to only 46.6% of their rural counterparts which is in line with earlier studies. But they also observe different relationship with other determinants such as age and religion and a positive relationship between a woman’s educational attainment with the three broad categories of antenatal care visits, place of delivery and tetanus toxoid injection.

Other authors like [13, 24] take a different approach to studying how health systems factors influence utilization of maternal health services by doing a diverse four-country comparison of which two countries are low income and two are middle income countries. In trying to close the gaps in prior studies on how key health systems characteristics affect maternal healthcare, they conducted a comparative analysis based on case studies of maternal health and health systems in Bangladesh, Russia, South Africa, and Uganda. They reviewed literature of published sources through international databases and other grey literature including policy documents, program reports, and student dissertations. They used the snowball sampling to fill gaps or clarify the available information and to suggest other relevant literature as well as DHS, and national statistics. For the case of Uganda, they reviewed 16 international publications/reports, 55 national reports and DHS for 1995 and 2001. The study found out that issues in the healthcare system influence access to and utilization of the same services as well as the quality of services provided and other maternal health outcomes. Specifically, they found out that the most important common system issues underlying maternal healthcare services were human resource structure systems, the blend of public and private sector service provision and the changes that resulted from the health sector reforms in respective countries.

Scholar [24, 25] argues that while skilled attendance (delivering with a doctor or midwife) and technical intervention are globally recommended as critical for reducing maternal mortality, maternal health outcomes for births that take place in hospitals still get affected by the systems in which they occur. A study by [26] shows the relationship between maternal mortality and skilled attendants at delivery in a number of countries and found that it was not a simple linear correlation. They identified other divergent factors such as the contribution of other medical staff who may be well trained and skilled as attendants.

It should be noted that whereas the study by [13, 27] identified common themes in the health service systems, they recommended that a great deal is dependent on the local context of each country. The extent to which a skilled attendant can do their job is dependent on the broader systems in which they operate. For instance, some areas with very low levels of skilled attendance may see lower mortality rates than in other regions, which score higher on this indicator because of the ability to reach emergency facilities as well as the quality of emergency care in dealing with life threatening complications.

A study by [4] is an example of an innovative study into determinants of maternal health services utilization in Uganda. Using the 2011 DHS data, they categorize the outcome variable into three packages namely: the ideal or desirable package, the moderate package and the undesirable package. In the ideal package, women attended at least four antenatal visits assisted by skilled personnel, delivered in a health facility and took their first post-natal check-up within 2 days following delivery. In the moderate package, women received less than four antenatal visits, had supervised delivery from a health facility and received postnatal care. In the last package, women did not have antenatal visits, did not use a health facility for delivery and had no post-natal care. A multinomial logistic regression is undertaken to analyze the contribution of various predictors of maternal healthcare services utilization package. They applied the Andersen’s Behavioural Model of Health Services Utilization for guidance in the selection of covariates in the regression model. They found that utilization of maternal healthcare services varied greatly by demographic and socio-economic characteristics. Key among them was education and region of residence that significantly influenced the utilization of the ideal package of maternal health services, and not the moderate services package. It is posited that holding other variables constant, having secondary (more education) increased the utilisation of the ideal package of maternal health services.

In regard to the impact of region of residence, the study found out that women who lived in regions outside Kampala, Uganda’s capital, were less likely to utilize the desirable package of maternal health services while those from the richest households were more likely to utilize the desirable maternal health services package. Other residual factors included residing in rural areas, being Moslem and being married which all reduced a woman’s chances of utilizing moderate maternal health care services. This study omitted variables deemed to cause heterogeneity such as cultural factors. Different societies especially in rural Uganda have strong cultural beliefs that women can handle births by themselves at home or with local unskilled birth attendants which might have had an impact on the findings of this study. The health services utilization models address a broad spectrum of factors which this study did not cover. However, the study concludes that enhancing utilization of desirable maternal healthcare services requires increased investment in secondary education at the community level, and strengthening of the health systems and improvement of quality of care.

Scholars like [4] scaled down the determinants of MHC services in developing countries to 3E’s (Economic status, Empowerment and Education). They pick DHS data for 31 countries and conduct separate logistic regression models for multiple countries on the use of antenatal services, contraceptives and skilled birth attendance. The study finds that women with gaps in the 3E’s across 31 countries of which 21 are in Africa were less likely to use maternal health services than those without gaps. Authors [4] further undertake a meta-analysis to obtain the pooled adjusted odd ratios and their confidence intervals for women in 31 countries and a sub-analysis for African countries. The results obtained from the 31 countries still match those from the 21 African countries which suggests the significance of the 3Es for women everywhere else. For instance, they find out that the odds of the poorest 20% compared to the richest 20% of women are 74%, 84% and 94% lower for use of modern contraceptives, having four or more antenatal care visits and skilled attendance at delivery, respectively.

The authors also find a high pattern of association for gaps in education where women who completed primary education are five times more likely to have had a skilled birth attendant at delivery and three times more likely to have at least four antenatal care visits respectively than those who did not complete primary education. They suggest that efforts to lower maternal mortality will fail in the absence of basic maternal healthcare services, which in turn are unlikely to become available without pro-poor health policies. If the assumption that expanding quality services may increase women’s motivation to use these services is reasonable, then women who are equipped with the 3Es are more likely to take advantage of the improved health systems.

## Maternal Health Care utilization—women and other health outcomes

The impact of education on the utilization of maternal healthcare services cannot be studied in isolation. Access to maternal healthcare services does not only help in averting maternal deaths among expecting mothers but it also has a strong bearing on a wide range of other equally important health indicators such as infant mortality, fertility and life expectancy. These indicators are still a persistent burden to most of the developing countries across the world.

To begin with, we address one of the most recent studies that examines the relationship between maternal healthcare utilization and infant mortality. While [28] tests the hypothesis that optimum utilization of maternal healthcare services during pregnancy, at delivery and after delivery reduce the rate of infant mortality in Ethiopia. Using the multivariate logistic regression model, they find that infant mortality was highly associated with multiple births, birth spacing, pregnancy losses related to miscarriages and abortion or stillbirth, child deaths, and maternal healthcare utilisation. The main finding is that infant mortality reduces by approximately 66% among mothers who highly utilise maternal healthcare services compared to those that do not.

The findings of [28] are an additional evidence to the studies by Cardwell [29]. All these studies posit that the ability to avert complications that lead to miscarriages and stillbirths as well as enhance fertility practices that promote child spacing and limit unwanted pregnancies begins with accessibility to maternal healthcare services. Coming back to Uganda, the country has had a challenge of short birth spacing, still births, abortion on top of high fertility rate of more than five births per woman as at 2016. This trend has only worsened the rates of maternal deaths and short life expectancies [16]. According to the World Bank as at 2019, the infant mortality rate stands at 33.4 per 1000 births while life expectancy is at 63 years. The combination of high maternal and child deaths coupled with high fertility rates has persistently kept the life expectancy of mothers and the general society for developing countries far below that of the developed world.

## Education and Maternal Health Care utilization

To begin with, the study bundles a few studies that examine how education affects maternal healthcare utilization by changing the maternal health seeking behavior of expecting mothers. Authors like [30–32] all agree and conclude that education changes the behavior and attitude of expecting mothers towards utilization of maternal healthcare services. These studies find that educated women are more likely to receive at least 4 antenatal visits-taking the first visit within first trimester, visit skilled health professionals, take 2 or more than 2 Tetanus Toxoid (TT) doses, deliver in health facility and receive postnatal visit within 42 days of delivery than the women who are not educated. However, most of these studies point out two important issues.

The above authors conclude that the higher the level of education attained, the higher the chances that women will change their attitude towards maternal healthcare utilization including changing their maternal healthcare seeking behaviors. For example [30] using cross sectional DHS data find that female education creates different levels of maternal health-seeking behaviour, with the higher-level education of post-primary/secondary and tertiary education being more pronounced than primary level education. They conclude that focus should be put on increasing female education beyond primary and secondary level in a bid to improve maternal health-seeking outcomes. Secondly, most of the existing studies run short of isolating the impact of education from other equally important variables. For instance, using a binary logistic regression model [4], find a relationship between maternal education and utilization of MHC services in India. However, the analysis reveals a significant variation in the likelihood of utilising all the MHC services among the higher level educated women and the uneducated. But when they adjust other demographic and social variables with education, the adjusted odds ratio becomes lower than the unadjusted odds ratio. This indicates that it is not only education that affects utilisation of maternal healthcare services but also other factors in society. Such factors may include maternal age, age at marriage, religion, wealth index, place of residence, exposure to mass media, and region. Failure to isolate the impact of education leaves a gap for further research particularly for Uganda, a country that has undertaken critical reforms in the education sector including UPE, Universal Secondary Education (USE), Affirmative action for a girl child joining any public university.

It is from the above shortcomings that we identify another empirical approach. Researchers like [9, 10, 14, 17] examine the impact of education on maternal healthcare utilization by identifying a different source of exogeneity using the RDD method. She uses a compulsory schooling reform in Peru that increased mandatory years of initial schooling from 6 to 11 years. The study examines how exposure to such a reform changed the behavior of expecting mothers regarding the ability to identify complications during pregnancy including detectable risks such as blood pressure and hemorrhaging; fertility practices such as use of contraceptives, occurrence of unplanned pregnancies and hence abortion; use of antenatal services, number of visits and other potential mechanisms such as cognitive skills arising out of literacy, income of households/wealth and autonomy in decision making.

In regard to her results, the author finds out from the first stage regression that women who were exposed to the reform had an average of 0.32 more years of formal education than women who were not exposed, making the reform a valid instrument. She then regresses pregnancy complications on the predictions from the first stage and finds out that increasing women’s education by a year would reduce their probability of complications such as fever accompanied by vaginal bleeding during pregnancy by 1 percentage point and the probability of fever after birth by 2 percentage points (p < 0.05). In the context of Peru, the effect can be interpreted as a 1 percentage point decrease in fever with vaginal bleeding during pregnancy being equivalent to a 25% decrease for Peru standards and a 2-percentage point decrease in fever after birth is equivalent to a 29% decrease compared to the average woman respectively in Peru. The same relationship is observed with how education affects fertility practices and antenatal healthcare utilisation.

## Universal Primary Education (UPE) in Uganda

Implementation of UPE across many countries came with a number of benefits as discussed in the structure of the Policy that was introduced in Uganda effective January 1997 [33]. The Government of Uganda eliminated user fees for primary pupils, including indirect fees through Parent Teacher Associations (PTA) and uniforms, for a maximum of four children per household of which two had to be girls. Before introduction of the policy, direct and indirect school fees made up more than 50% of school income. Implementation of UPE was supported with a strong dissemination campaign that was highly skewed towards keeping girls in school by focusing on girls’ education and the negative impact of early marriages. For this study, we use 1997 as the year of discontinuity for the analysis.

Introduction of UPE caused a 68% jump in school enrollment, from 3.1 million in 1996 to 5.3 million in 1997 [18, 20]. Accordingly, enrollment rates were much more pronounced among the poor than the non-poor after the introduction of UPE, thus narrowing the enrollment gap between the poor and non-poor. But most importantly, enrollment rates for girls increased significantly with primary school attendance among girls of 6 to 12 years old growing from 59.7% in 1992 to an estimated 83.2 and 91% in 1999 and 2004 respectively.

Literature from [20] provides a more systematic assessment of the UPE Programme by complementing data from the 1992 Uganda Integrated Household Survey collected from 10,000 households with information from the 1999/2000 Uganda National Household Survey (UNHS) collected from 6000 households. The sample age was between 6 and 8 years for both surveys. Using a combination of descriptive evidence and repeated cross-section estimates, the author finds that UPE greatly reduced the wealth bias that had characterized access to primary education in 1992. But most importantly and related to the question in this study, UPE helped to reduce gender gaps and establish gender equality by increasing girls’ access to primary education; and reduced the incidence of cost-related drop-outs from primary school. While [10, 34] confirms that UPE helped the poor and marginalized to access primary education with the most dramatic increase being for girls aged 6 to 8 years benefiting the most.

The increase in access notwithstanding, the UPE policy in Uganda as well as in other SSA countries has been criticized for improving access to primary school at the expense of deteriorating school quality [4, 16]. It should however be noted that this study examines only issues related to school enrollment and access not the quality of the education received from the UPE programme.

## Implications for the approach

Extensive literature attributes increased access to and utilisation of maternal healthcare services to education. However, as highlighted by [4, 16], the key challenge in establishing the causality is the inability to isolate the impact of education on the attitude and behaviour of expecting mothers towards seeking maternal health services. Unless the impact of education is exogenously isolated, in this case using the RDD approach, it remains difficult to establish whether the change in maternal healthcare utilisation is entirely attributable to the effect of education or to other related factors including but not limited to the trend in economic growth, age of women, size and incomes of households or wealth, region or place of residence, religious background, marital status among others.

Vitally, the regression discontinuity takes advantage of an exogenous source of variation, the UPE Policy in 1997 that waived all direct and indirect school attendant costs. Some factors such as the number of siblings and income may affect both the years of education and utilisation of healthcare services, there is a possibility of downward bias in the OLS model estimates on the effect of education on use of maternal healthcare services. The RDD approach is free of such bias because it employs an exogenous threshold that affects years of schooling in year 1997. We learn from the analysis that the effects of education enhance women’s cognitive skills, material resources and autonomy, which in turn affect their health outcomes as highlighted by [6, 23, 25, 35, 36].

## Data and methodology

### Study area

**Fig. 1:**
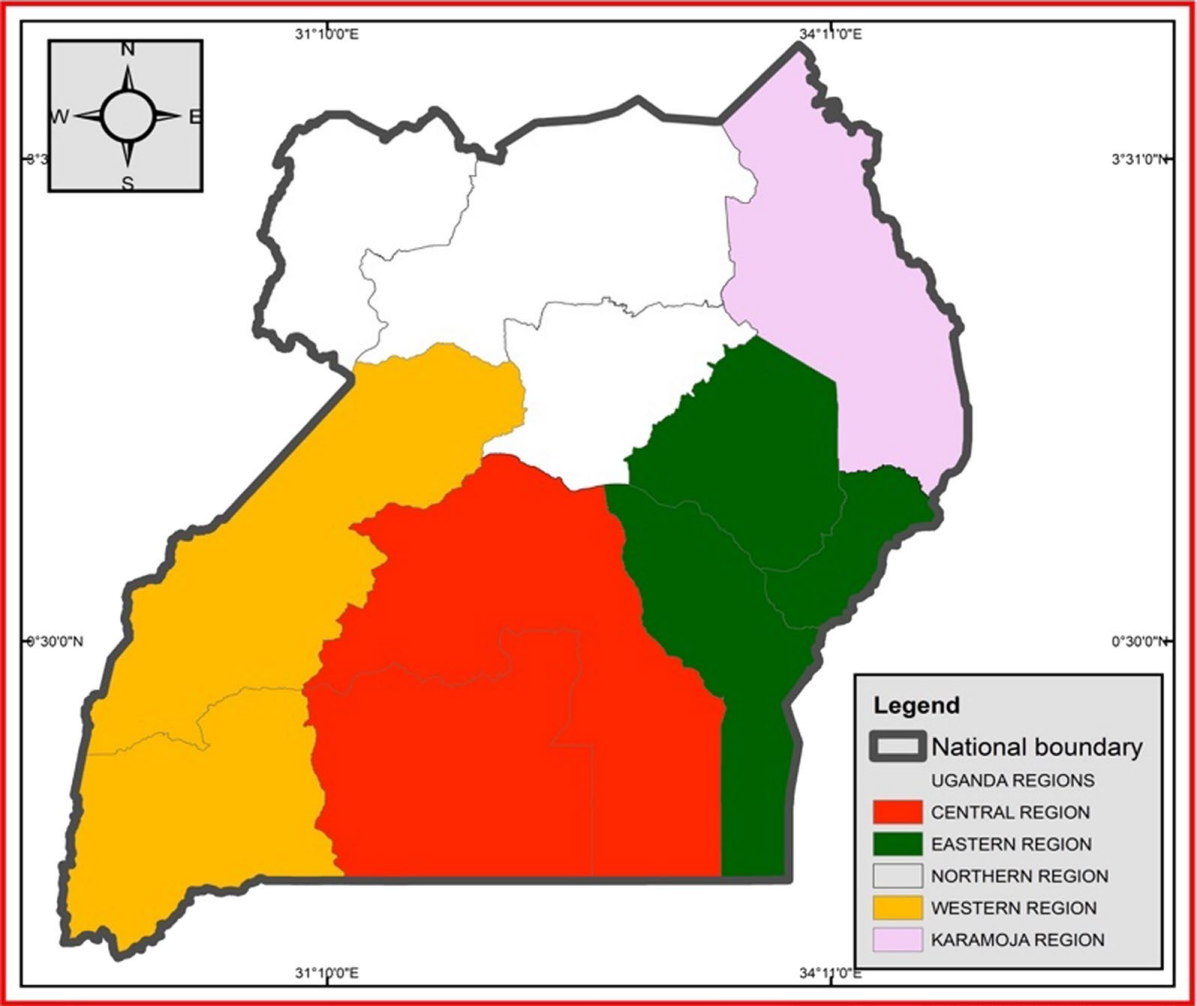
Map of Uganda showing the regions of the study

The map in Fig. 1 shows the regions where data was collected. The central part of the country, based on the national data, is the most literate region followed by Western, then Eastern, Northern and least is Karamoja region. The northern region had a civil war spearheaded by Joseph Kony for almost 20 years since 1986 to 2006 and therefore they were somehow affected by the conflict in terms of having access to basic social services, education inclusive. On the other hand, Karamoja region is a nomadic pastoral region where children keep on wandering from one place to another looking for pasture and water for their animals and hence do not spend quality time studying.

### About the data for this study

Uganda is a suitable country for this study because of its past trends in MMR and other related health indicators such as infant mortality, fertility, life expectancy. The MMR has been falling since the year 2000 from 580 deaths out of 100,000 live births to 343 out of 100,000 births in 2018 which is still high compared to the global target of 70 deaths per 100,000 live births [16]. Regarding the data for this study, we use a national representative cross-sectional data from the continuous Demographic and Health Survey (DHS) of 2006 and 2011 collected from Uganda by the DHS in collaboration with the Uganda Bureau of Statistics (UBOS). The data was formally requested for from Demographic Health Survey (DHS) Website. For this study, we take keen interest in the individual questionnaire that is answered by women in households, aged 15 to 49 on key demographic issues ranging from maternal healthcare, age, education and child health.

**Fig. 2:**
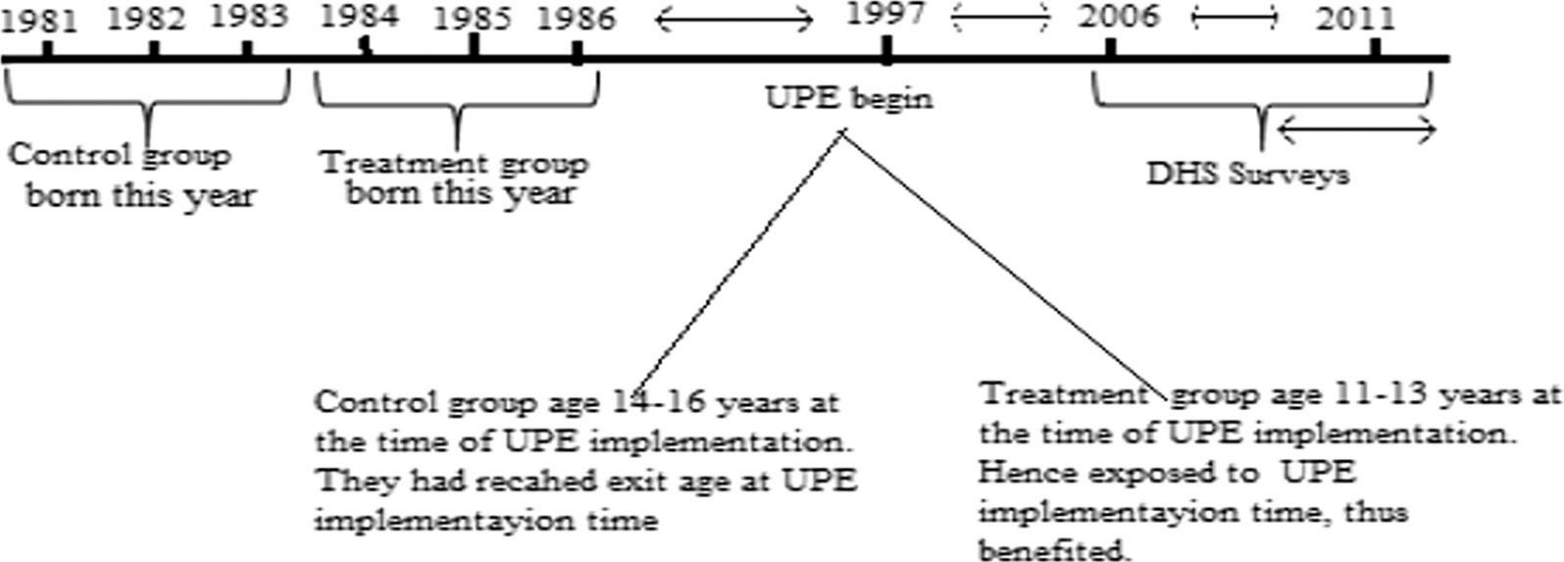
Visual representation of the clusters

For the final sample used in this study, it should be noted that we restrict the sample to 52,024 women who represented the cohort age between 11 and 16 years for the birth years of 1981 to 1986. This is a representation of respondents who were aged just above and just below the average age of completing primary school as at 1997 when the UPE programme started. Figure 2 is a visual representation of the timeline of the events for the two groups; 11 to 13 years who were just below primary level exit age in 1997 and 14 to 16 years who were just above the age of primary school exit age in 1997. All respondents in the sample are therefore between the ages of 25 and 30 years given that the survey was undertaken in 2006 and 2011.

Additional observations are dropped due to missing data while other observations particularly religion and region are merged for example since the Seventh Day Adventist (SDA) are less than 1% of the entire religion, we included them in the category of “others”. In seeking to categorise the data for ease of identification, some sub regions were merged for example the Western and South Western sub regions were merged into Western region while the North and North East sub regions were merged to form the Northern Region.

Table 1 represents the summary statistics from the key variables that are assumed to affect the education of an individual. In terms of religion, Catholics represent 44.3% followed by Protestants at 30.31%, then Muslims at 11.58%, Pentecostal at 8.19% and lastly Others at 5.63%. The category of others represents all the minor religious sects across the country. This is not a surprise as the Uganda Census (2014) indeed supports these results where the distribution of religion in Uganda matches the findings of this study and has a direct impact on the education sector. Indeed, most public schools derive their foundation from churches and mosques. The next variable that is associated with education is the region and findings reveal that 26.74% of the respondents were from the Central region, 6.23% came from the Karamoja region, 27.76% were from Northern Uganda, 15.04% were from Western region, and 24.23% were from Eastern Uganda. These findings are also not surprising as they are associated with the findings from Uganda Census results of 2014. Residence is also assumed to be highly associated with education, and in this case, the study categorizes the residence into Urban (13.41%), Semi-urban (57.01%) and Rural (29.58%). In the study it was also important to consider the sex of the household head and in this regard, households headed by males represented 71.02% while those headed by women represented 28.98%. The number of years of education averagely represented 4.82 with a standard deviation of 4.41 and a range of 0 to 18 years implying that some respondents had never gone to school for formal education at all. The study also considered Wealth index which is a proxy to household wealth. The study findings revealed 68.52% that on average, wealth index was − 12,661.80 implying abject poverty in the majority of the households. The figure of 409,545 implies that there are rich families as well. Lastly, the number of siblings in this study was on average 6.37 with a standard deviation of 2.82. This result also makes sense given Uganda’s fertility rate as at 2016 of more than 5 children per woman [3]. Furthermore, the range of 1 to 15 children is pretty high for a majority of the families being poor as seen from the findings and hence this is expected to affect the years of education as there will be struggle for few resources to spend towards education.

**Table 1:** Summary statistics for the key study variables

Categorical variables	Control		Treatment		p-value
	N	Proportion (%)	N	Proportion (%)	
Religion					
Catholic	5028	37.45	13,692	47.49	
Protestant	3836	28.57	8971	31.11	
Muslim	1249	9.3	3643	12.63	< 0.001
Pentecostal	1663	12.39	1797	6.23	
Others	1650	12.29	731	2.54	
Residence					
Urban	1528	11.38	4139	14.35	
Semi-urban	9943	74.06	14,151	49.08	< 0.001
Rural	1955	14.56	10,544	36.57	
Region					
Central	4529	33.73	6772	23.49	
Karamojong	299	2.23	2332	8.09	
North	3893	29	7839	27.19	< 0.001
Western	1704	12.69	4651	16.13	
Eastern	3001	22.35	7240	25.11	
Sex of the household head					
Male	11,946	72.94	24,999	70.13	0.161
Female	4431	27.06	10,648	29.87	0.074

Continuous variables	N	Mean ± SD	N	Mean ± SD	
Age	16,377	24.66 ± 2.09	35,647	21.87 ± 2.13	0.091
Number of years of education	35,647	4.481 ± 4.87	16,377	6.27 ± 2.87	< 0.001
Number of siblings	16,377	6.40 ± 2.75	35,647	6.35 ± 2.85	0.844
Wealth Index	16,377	− 16,413.1 ± 89,111.38	35,647	− 10,938.4 ± 91,277.72	0.033
Observation = 52,024					

**Fig. 3:**
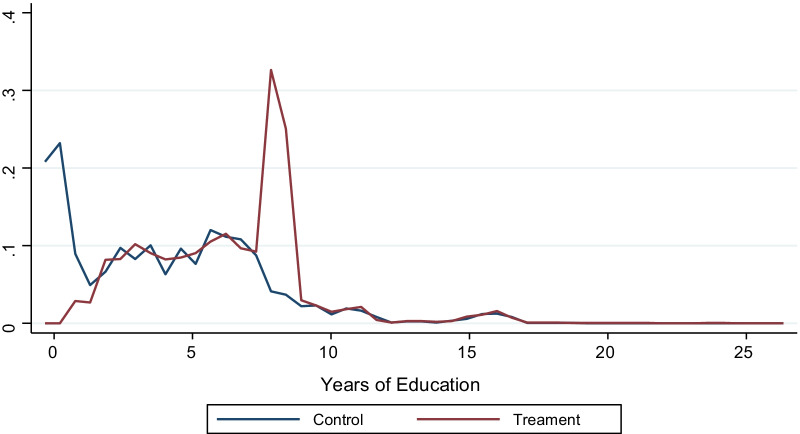
Kernel distribution of years of education

Generally, the treatment group spent more years at school than the control as shown in Fig. 3 and this is confirmed by the results in Table 1.

### The models used

In this section, the study seeks to explain the methodology that was used in explaining years of schooling as the study controls for other factors that are assumed to affect education. The study uses several models to achieve this, that’s the Ordinary Least Squares (OLS), the Two Stage Least Squares (2SLS), the Regression Discontinuity Design (RDD) and fixed effect model to check for any kind of heterogeneity in the data. The OLS for the first stage model was used to predict the years of education controlling for a number of factors like region, religion, residence age, sex, wealth and number of siblings in a family as is in [34, 37]. The study then uses the predicted values from the first stage model to determine its effect on the maternal health utilization. Maternal health utilization is categorized into three broad themes namely; antenatal utilization, complications during pregnancy and fertility practices. In the second stage model the study uses age as an Instrument Variable. The study then uses the RDD model to understand the effect of introduction of free education to the cohort group. Lastly, the study fit a fixed effect model to control for kind of heterogeneity in the data. In this particular case the study uses region since UPE was introduced at the same time in all regions in Uganda but because of the civil war in northern Uganda, there was disruption in implementation of the programme in other regions of the county particularly Northern Uganda.

### The model for effect of years schooling-First stage model

In this case the study uses the Ordinary Least Squares (OLS), controlling for region, religion, residence age, sex, wealth and number of siblings in a family as they are assumed to affect someone’s years of schooling. The response variable for the years of schooling being continuous, then the OLS model was plausible. The model is as specified as below:

1 $$Edu{c}_{i}={\beta }_{0}+{\beta }_{1}Religion+{\beta }_{2}Region+{\beta }_{3}Residence+{\beta }_{4}Age+{\beta }_{5}Sex+{\beta }_{6}Wealth+{\beta }_{1}Siblings+ {\varepsilon }_{i}$$

where $Edu{c}_{i}$ is the years of education with $i=\mathrm{1,2},3,\dots ,n$, ${\beta }_{0}, {\beta }_{1}, {\dots ,\beta }_{6}$ are the parameter estimates and the ${\varepsilon }_{i}$ is the errors term which is assumed to follow a normal distribution and identically distributed. The independent variables are defined as below:

$$Residence \left\{\begin{array}{l}1=Urban \\ 2 =semiurban \\ 3=Rural \end{array}\right. \quad Religion \left\{\begin{array}{l}1=Catholic \\ 2 =Protestant \\ 3=Muslim \\ 4=Pentacostal\\ 5=Others \\ \end{array}\right.$$

$$Region \left\{\begin{array}{l}1=Central. \\ 2 =Eastern \\ 3=Northern \\ 4=Western. \\ 5=Karamoja \\ \end{array}\right. \quad Sex\, of\, household\, head \left\{\begin{array}{l}1= Male\\ 2=Female\end{array}\right.$$

Age is the respondents’ age in cohort group, Wealth is the wealth index while siblings is the number of siblings who live in the household.

### The Second Stage Least Squares (2SLS) model with an instrumental variable approach

The 2SLS follows from Eq. (1) where the study regresses the outcome ${Y}_{i}$ on the predicted values of ${D}_{i}$ (years of schooling). The empirical model can be precisely specified as below:

2 $${Y}_{i}={\beta }_{0}+{\beta }_{1}{D}_{i}+\dots +{\beta }_{k}{x}_{k}+{\varphi }_{i}$$

where ${Y}_{i}$ is the outcome variable, ${x}_{k}$ is the Instrumental Variable (IV) and in this case age is controlled for in the second stage model and it is regarded as the IV variable since it is an endogenous due to the fact that it changes with years of schooling. ${{\beta }_{0} ,\beta }_{1},{\beta }_{k}$ are regressors and ${\varphi }_{i}$ is the error terms which is assumed to follow a normal distribution and identically distributed.

The model can be rewritten as:

3 $${Maternal}_{i}={\beta }_{0}+{\beta }_{1}{Schooling\_predicted}_{i}+\dots +{\beta }_{k}{Age}_{k}+{\varphi }_{i}$$

For ${Maternal}_{i}$ implying maternal health care utilization and ${Schooling\_predicted}_{i}$ is the predicted years of schooling and ${Age}_{k}$ is the age of the respondent taken as my IV.

### Regression Discontinuity Design (RDD) model

**Fig. 4:**
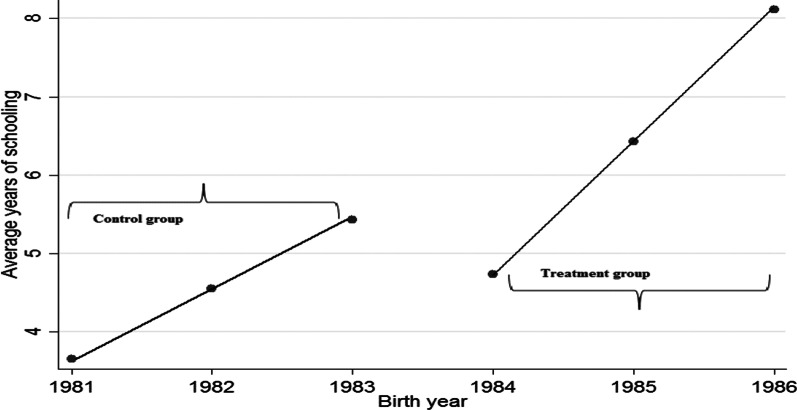
Line graph for the years of education versus birth years

In this section, the study sought to find out the effect of the introduction of free primary education on the years of schooling. Since the introduction of free primary education in 1997, the study traces back the children who would have benefited from it, represented by a group born between 1984 and 1986 forming my treatment group. Then those who were born between 1981 and 1983 are considered to have surpassed the primary level completion age and hence they form the control group. To be able to demonstrate this, the study uses the RDD model which estimates treatment effects in non-experimental settings and can provide a causal estimates of treatment effects. In this model, the study assumes that introduction of free education induced a discontinuity in the increase of the number of years of schooling on those who benefited at that time. This discontinuity should be exogenous and independent of the unobserved characteristics that affect the child’s education. Therefore, creating a good setting for RDD model using year of schooling and birth years as the rating variables as shown in the scatter in Fig. 4 of the average year of schooling and birth years.

The study also explored the years of evolution of schooling for the cohort group as in Fig. 3. Indeed, the years of education between the control and treatment increased over the birth years implying there was mass enrolment and also many years in school since the implementation of free education. Furthermore, from 1981 to 1986 most parts of Uganda experienced political instability and majority of the people were extremely poor. After 1986, the new government came in with relatively a high level of social, political and economic stability with new reforms which saw more children going back to study. Hence a sharp rise in the level of years of schooling.

The RDD model allows us to compare those individuals assumed to have benefited from the free education and those who never benefited from the free education. This normally generate a local treatment effect, for those individuals at the bottom of those who were affected by the introduction of free education. In this model the study employs one cut off point with one running variable birth years. The RDD implements a set of regression-discontinuity estimation that are thought to have very good internal validity, for estimating the causal effect of some explanatory variable (called the treatment variable) for a particular subpopulation, under some often-plausible assumptions. Therefore, the empirical model is as stated below:

4 $$Edu{c}_{i}= \widehat{\gamma }UP{E}_{i}+f\left({X}_{i}-c\right)+{\varepsilon }_{i}$$

where $\widehat{\gamma }$ is the estimate of the local average treatment effect of person’s birth years affected by the introduction of free education, $UP{E}_{i}$ is a dummy of whether one benefited from free education (treatment coded as one) and never benefited from free education (control group coded as zero), ${X}_{i}$ is the rating or the forcing variable which represents the birth year of the cohort respondents, *c* represents the cut-off of the introduction of the free education. The functional form of the rating variable is normally assumed to be important in RDD model as it controls for any heterogeneity in the cohort at around the cut-off point. The study therefore, estimates the effect of introduction of free education using a fuzzy RDD using least squares approach, with a dummy variable for whether someone benefited or never benefited from free education.

### Fixed effect model to control for heterogeneity in the data

The fixed effect model was used to control for exogenous and unobserved time-invariant heterogeneity variables between the 2-year block (2011 and 2006).

Consider the panel regression model and the study applies probit model.

5 $${P(Y}_{it}=1)={\beta }_{0}+{\beta }_{1}{Schooling}_{it}+{\beta }_{2}{region}_{i}+{u}_{it}$$

where the ${P(Y}_{it}=1)$ is the probability of maternal health utilization, ${Schooling}_{it}$ is the years of schooling and ${region}_{i}$ are unobserved time-invariant heterogeneities across the regions $i=1,\dots ,5$. We aim to estimate ${\beta }_{1}$, the effect on ${maternal}_{i}$ of a change in ${Schooling}_{it}$ holding constant ${region}_{i}$. Letting ${\alpha }_{i}={\beta }_{0}+{\beta }_{2}{region}_{i}$ obtaining the following model.

6 $${Maternal}_{it}={\alpha }_{i}+{\beta }_{1}{Schooling}_{it}+{u}_{it}$$

Having individual specific intercepts ${\alpha }_{i}$, $i=1,\dots ,n$, where each of these can be understood as the fixed effect of entity *i*, this model is called the fixed effects model. The variation in the α_i_, $i=1,\dots ,n$ comes from the ${region}_{i}$.

### Robustness checks

For any model to stand any test, it is always good to check for its robustness. The study was therefore subjected to model robustness checks. First the OLS model against its assumption of normality and heterogeneity of the error terms. The RDD model was subjected to sensitivity analysis by trimming off 5% of both the top and bottom cohort samples. Then we also implement the model with various degree of polynomial to see how sensitive the outputs are from the rating variable.

## Results

In this section, the study discusses the results from the different models that were fitted. We discuss the results from OLS in Table 2, the 2SLS in Table 3, the RDD Table 4 and fixed model in Table 6.

**Table 2:** OLS model estimates

Variable	Coef	Std. err	t	p > t	[95% Conf Interval]
Religion					
Protestant	− 0.00	0.05	0.00	0.999	− 0.10, 0.10
Muslim	0.15	0.07	2.24	0.025**	0.02, 0.27
Pentecostal	1.28	0.08	16.64	0.000***	1.13, 1.43
Others	− 4.50	0.09	− 47.44	0.000***	− 4.69, − 4.32
Region					
Karamoja	− 2.02	0.09	− 22.78	0.000***	− 2.19, − 1.84
North	0.17	0.06	2.78	0.005***	0.05, 0.28
Western	0.71	0.07	10.34	< 0.001***	0.58, 0.85
Eastern	0.99	0.06	15.99	< 0.001***	0.87, 1.11
Residence					
Semi-urban	1.14	0.07	16.83	< 0.001***	1.01, 1.28
Rural	− 0.14	0.07	− 1.93	0.054*	− 0.28, 0.00
Age	0.58	0.01	49.38	< 0.001***	0.56, 0.60
Sex (Female)	0.13	0.04	3.06	0.002***	0.05, 0.21
Wealth Index	0.00	0.00	6.44	< 0.001***	0.00, 0.00
No of siblings	− 0.04	0.01	− 6.09	< 0.001***	− 0.05, − 0.03
_Cons	− 8.43	0.27	− 31.41	< 0.001***	− 8.95, − 7.90

Source	SS	df	MS	Number of obs	52,024
				F(15,5204)	251.11
Model	15,170.66	15	1011.38	Prob > F	< 0.0001***
Residual	221,937.36	55,104	4.03	R-squared	0.64
				Adj R-squared	0.637
Total	237,108	55,119	4.30	Root MSE	2.0069

^***^p < 0.001, **p < 0.05, *p < 0.1

**Table 3:** Second stage model (2SLS) estimates

	Antenatal utilization		
	Knowledge of ovulation	Visited health facility	Insurance
Year of education	0.26*** (0.01)	0.030*** (0.01)	0.001 (0.004)
Constant	4.49*** (0.06)	0.58 *** (0.02)	-0.029 (0.016)
N	52,024	15,699	15,699
Wald chi2(1)	5265.24	30.93	2.50
p-value	< 0.0001***	< 0.0001***	0.1137

	Complications during pregnancy		
	Blood pressure	Anaemia	Intake of supplement (Iron)
Year of education	0.11*** (0.03)	2.34*** (.08)	3.14*** (.01)
Constant	0.01 (0.15)	− 6.35*** (0.42)	− 1.41*** (0.54)
N	2639	1764	1764
Wald chi^2^(1)	12.19	807.45	200.01
p-value	0.0005***	< 0.0001***	< 0.0001***

	Fertility practice				
	Postnatal checks	Breast feeding	Termination of pregnancy	Knowledge of family planning	Number of children
Year of education	0.02** (0.006)	0.27*** (0.01)	− 0.04 *** (0.004)	0.34 (0.021)	− 0.14*** (0.010)
Constant	0.97*** (0.035)	1.60*** (0.05)	0.81*** (0.03)	3.55 (0.10)	3.42*** (0.053)
N	23,374	26,561	26,561	42,260	26,561
Wald chi^2^ (1)	9.30	768.25	70.10	255.39	209.64
p-value	0.0023***	< 0.0001***	< 0.0001***	< 0.0001***	< 0.0001***

^***^p < 0.001, **p < 0.05, *p < 0.1

**Table 4:** Model estimates of the RDD

Estimating for bandwidth 0.8857					
Variable	Coef	Std. err	z	p >|z|	[95% Conf Interval]
Birth years	0.84	0.41	10.31	0.000***	0.13, 0.91
_cons	0.03	0.11	0.51	0.972	− 0.29, 0.41
N = 52,024					
Prob > F = < 0.0001***					
R-squared = 0.161					

^***^p < 0.001, **p < 0.05, *p < 0.1

In the first stage model, we adjust for religion, region, residence, age, sex, wealth index and number of siblings in the household. Religion greatly impacted on the years of schooling for example a unit increase in the number of Muslims led to 0.15 increase in the years of schooling as compared to the Catholics counterparts. Then being a Pentecostal also had a positive impact on the level of education as a unit increase in someone’s being a Pentecostal led to 1.28 increase in years of schooling. However, others as a religious category led to a reduction of years of schooling by 4.5. This can be attributed to, the different belief other religions have towards education as some of them claim that through education, they are being initiated to satanism (Table 5).

**Table 5:** Robustness checks

Variable	Coef	Std. err	z	p >|z|	[95% Conf Interval]
Birth year	0.14	0.34	6.31	0.000***	0.12, 0.87
Birth year squared	0.10	0.32	4.11	0.031**	0.121, 0.678
_cons	0.12	0.11	0.21	0. 251	− 0.13, 0.51
N = 52,024					
Prob > F = < 0.0001***					
R-squared = 0.163					

^***^p < 0.001, **p < 0.05, *p < 0.1

The region is also an important factor in the model and those respondents who studied from Karamoja region had reduced years in school that’s 2.02 as compared to central region. However, for other regions, they had a positive effect on the years of schooling with 0.17, 0.71 and 0.99 for north, western and eastern respectively increased effects on the years of schooling as compared to the central region. The findings from Karamoja region can be attributed to the pastoralism nature where children keep wandering from place to place looking for pasture and water for their animals and hence less time to sit in the classrooms to study. The place of residence also had an impact on the years of schooling in the semi urban residence compared to the urban residence had 1.14 increased chance of schooling.

However, for the rural residence compared to the urban residence had − 0.41 reduction in the years of schooling. This is common phenomenon in Uganda where very deep in the rural setting the parents don’t care very much to ensure that their children study. Hence most of them end up dropping out of schooling even before completing primary school [4]. Age and sex also had a positive impact on increasing the years of schooling with an effect of 0.58 and 0.13 for age and sex respectively. The wealth index also had positive effect in the years of schooling and is directly related to someone’s level of income which makes him able to pay school fees. However, the number of siblings had a negative effect on the years of education with a value of − 0.04. This is attributed to resources needed to look after the children at home and other family members.

In the second stage model outcomes were classified as antenatal utilization, complications during pregnancy and fertility rates. The antenatal utilization consists of: Knowledge of ovulation, visited health facility, and access to insurance. While complication during pregnancy consists of: Blood pressure, Anaemia and intake of supplement like iron. Lastly, fertility practices consist of: Postnatal check, breast feeding, termination of pregnancy, knowledge on family planning and number of children. For the variables on antenatal utilization, those women who had spent more years in school had much knowledge on family planning as such, they would always frequently visit health facilities and some of them had taken a step to have life insurance to cater for any health-related challenges.

For complication during pregnancy factors, those women who had spent more years at school would always go to check for blood pressure and also anaemia. This is an important factor for timely intervention as it has been known that such non-communicable diseases like pressure is among the leading causes of death in Uganda [8, 9, 17].

Lastly, for fertility practices, those women who had spent much time at school had higher chances of going for postal natal checks, do breast feeding and knowledge of family planning. They also had a reduced chance of termination of pregnancies since they know the dangers associated with such practices as well as reduced number of children.

**Fig. 5:**
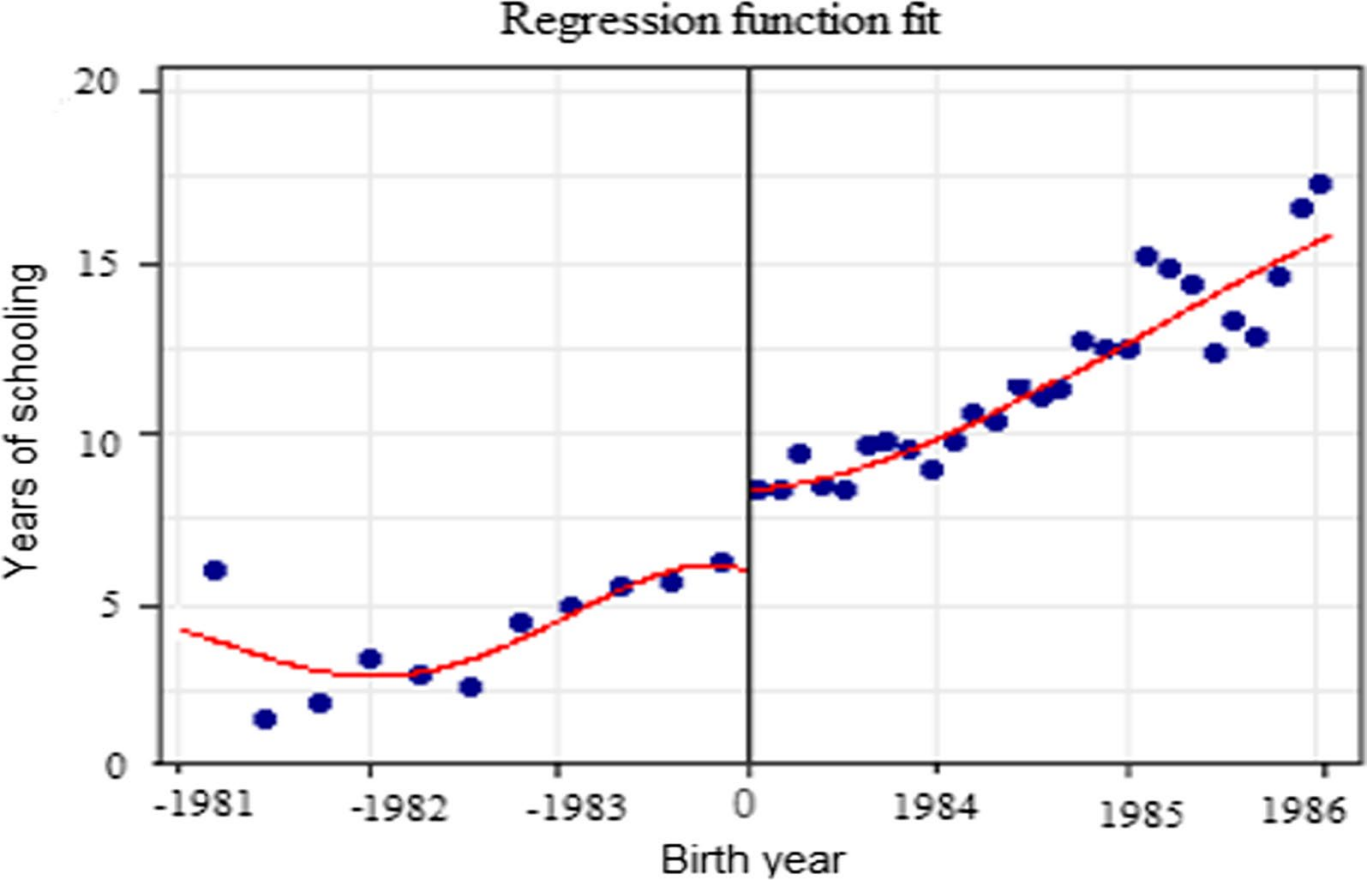
Discontinuity plot for years of education with respect to birth year

The estimates from the model are positive and significant implying that the years of schooling increased with the increase in the respondent’s birth years in fact by 0.84 years. This can also be confirmed by the discontinuity plot in Fig. 5 as there was a big jump between the time if implementation of free education.

The robustness check employed was based on squaring the values of age to see whether the conclusion on age will still remain significant. Since the birth year remained significant, then the model can be relied on to predict the years of schooling.

In Table 6, the study controls for the fixed effects in the model by fitting a fixed effects model on the outcome variable of the maternal health outcomes. These are driven from the second stage model. The study controls for the region as this is assumed to be heterogeneous as a result of civil war in northern Uganda by the time free education was introduced. Factors related to maternal health utilization such as knowledge of evaluation, visiting the maternal health and insurance all had positive influence on education after controlling for region. Indeed, region knowledge of evaluation, region 1.35 (exp (0.31)) odds of increased maternal health utilization. Similarly, visiting health facilities for antenatal advice and checks also had a positive impact on education that’s odds ratio of 3.71. Then embracing of insurance services, the odds ratio was, 1.12. Generally, the overall effect of region, northern and Karamoja region for the three factors on the antenatal utilization was always negative throughout all the models and can be attributed to the civil war and the nomadic lifestyle for the North and Karamoja regions respectively.

**Table 6:** Fixed effects model estimates

	Antenatal utilization		
	Knowledge of ovulation	Visited health facility	Insurance
Year of education	0.31** (0.05)	1.31*** (0.11)	0.11** (0.05)
Region			
Karamoja	− 15.51** (0.084)	− 4.24** (0.01)	− 6.30** (0.04)
North	− 2.22*** (0.74)	− 1.03*** (0.24)	− 1.31*** (0.07)
Western	0.02 (0.29)	0.43** (0.20)	1.34 (0.09)
Eastern	− 1.06** (0.46)	0.15** (0.07)	− 1.12** (0.11)
N	52,024	15,699	15,699
LR chi^2^(1)	32.65	54.23	74.61
p-value	< 0.0001***	< 0.0001***	0.001**

	Complications during pregnancy		
	Blood pressure	Anaemia	Intake of supplement (Iron)
Year of education	0.13*** (0.03)	0.04*** (0.56)	0.78*** (0.11)
Region			
Karamoja	− 3.51*** (1.14)	− 5.90** (0.35)	− 1.46** (0.35)
North	4.76*** (0.07)	1.03*** (0.24)	0.64*** (0.06)
Western	1.32 (0.78)	1.03** (0.07)	0.14** (0.98)
Eastern	2.16** (0.46)	2.25** (0.47)	0.132** (0.32)
N	2639	1764	1764
LR chi^2^(1)	100.34	87.45	217.45
p-value	< 0.0001	0.0001***	< 0.0001***

	Fertility practice				
	Postnatal checks	Breast feeding	Termination of pregnancy	Knowledge of family planning	Number of children
Year of education	0.02** (0.006)	0.27*** (0.01)	− 0.04 *** (0.004)	0.34 (0.021)	− 0.14*** (0.010)
Region					
Karamoja	− 7.08*** (0.41)	0.31** (0.05)	7.02** (0.44)	− 4.11** (0.03)	− 0.36** (0.11)
North	1.46*** (1.17)	2.04*** (0.04)	0.44*** (0.01)	0.43*** (0.02)	0.22*** (0.11)
Western	0.32 (0.08)	1.13** (1.17)	0.34** (0.08)	0.03** (0.17)	0.35** (0.08)
Eastern	1.26** (0.06)	4.35** (0.07)	0.42** (0.02)	0.25** (0.07)	0.14** (0.55)
N	23,374	26,561	26,561	42,260	26,561
LR chi^2^(1)	50.25	68.11	105.21	55.04	98.03
p-value	< 0.0001***	< 0.0001***	< 0.0001***	< 0.0001***	0.0008***

^***^p < 0.001, **p < 0.05, *p < 0.1

For complication during pregnancy models, such as blood pressure checks, anaemia and, intake of supplements particularly iron, also a positive effect on education. The mothers who had taken iron supplements, had increased odds for example of 1.34, 1.04 and 2.18 respectively for blood pressure checks, anaemia and intake of supplements. For Karamoja region, the effects of complications during pregnancy were negative.

Lastly, the study looked at the fertility practices such as postnatal checks, breast feeding, termination of pregnancy, knowledge of family planning and number of children. Controlling for heterogeneity due to region also had showed that postnatal checks increased by 1.02, breast feeding increased as result of education, with odds ratio of 1.31, termination of pregnancy reduced by odds of 0.96, knowledge of family planning increased by odds of 1.41 and number of children and number of children reduced significantly by 0.86. Though still, this was not a uniform influence for all regions, as Karamoja region for some practice on fertility had negative effects for example postnatal checks, knowledge on family planning and number of children.

Generally, studies have showed that education can increased the utilization of maternal health significantly [5, 20, 23, 35]. Therefore, government together with its development, partners should invest more in educational and where necessary feed also the pupils so that they can stay in school for example for the case of Karamoja.

## Discussions

Since the launch of UPE in 1997, there has been some tremendous achievements such as an increase in the level of literacy. However, fewer studies in Uganda have been done to assess whether the increase in the education level has led to increase in the maternal health utilization. This study attempted to analyze whether free education that was introduced in Uganda since 1997 had had an impact on the maternal health utilization. Since it has generally been observed globally that beginning of the twenty-first century saw a witnessed significant declines in child mortality [36] and maternal morbidity and mortality worldwide. Education has been proved as one of the vehicles that can propel utilization of maternal health services [4, 16].

The analysis involved using a number of modelling techniques such as, OLS, 2SLS, RDD as well as fixed effect model to account for heterogeneity in the model due to time variation. Models involved controlling other factors that could affect the level of education (age, religion, marital status, sex, wealth, number of siblings, and current place of residence). Outputs from the models were used to generate the predicted value that were used in the second stage model. In the second stage model the outcome is the health care utilization verses schooling (predicted) as the predictor. The RDD model was used to check whether there was an increase in the years of schooling after the introduction of education [4]. Then lastly, fixed effects model was used to control heterogeneity factors due to region as it is known that other regions were not stable at the time of implementation of the free education. For women living in wealth households, mother’s age, region, religion, residence and sex appeared to be the influential factors, showing significant either negative and positive effects [10, 14], partner’s education [38, 39], women’s age [10, 14, 40, 41], access to health facility [34, 37], religion[41] and working status [38] are also important factors associated with the outcomes of interest. The predicted years of education in the subsequent model had an influence in the maternal health utilization.

## Conclusion and recommendation

The findings from the study suggest that the more years the woman spends at school, the more are the chances of maternal health update. The study also showed that the region of a respondent was an important factor in the study. For example, female respondents who are from Karamoja, region had less chances towards maternal health utilization. However, this is attributed to the long history of the civil war which occurred in the northern part of the country which made women not to access proper education. For Karamoja region it is as a result of nomadism where the teenagers keep on wandering from place to place looking for pasture for their animal and have less time to study [11]. Therefore, as an effort to improve professional maternal health care utilisation, there is need to focus on female education beyond primary level. Government should also undertake to improve community infrastructure and security across all regions and locations. Both government and donors can ensure universal access to professional maternal health care irrespective of the ability to pay.

## Data Availability

Data is available on the DHS website, and it was formally applied for.

## Abbreviations

ANC: Antenatal care
MDG: Millennium Development Goals
MHC: Maternal health care
MMR: Maternal Mortality Ratio
MTC: Mother to child
OLS: Ordinary Least Squares
PNC: Postnatal care
RDD: Regression discontinuity design
SGD: Sustainable Development Goals
SSA: Sub Saharan Africa
TT: Tetanus toxoid
UN: United Nations
UPE: Universal Primary Education
WB: World Bank
WHO: World Health Organization
2SLS: Two Stage Least Squares
